# Multivariate analysis of risk factors for predicting supplementary posterior instrumentation after anterolateral decompression and instrumentation in treating thoracolumbar burst fractures

**DOI:** 10.1186/s13018-015-0155-2

**Published:** 2015-01-28

**Authors:** Jiang Chen, Yu-Song Jia, Qi Sun, Jin-Yu Li, Chen-Ying Zheng, Jian Du, Chun-Xiao Bai

**Affiliations:** Department of Orthopaedics, Dongzhimen Hospital, University of Chinese Medicine, Beijing, 100700 China

**Keywords:** Thoracolumbar burst fracture, Anterolateral decompression, Posterior instrumentation, Multivariate analysis, Risk factors

## Abstract

**Background:**

Although anterolateral decompression and instrumentation has several advantages in treating thoracolumbar burst fractures, the risk factors for supplementary posterior instrumentation are still unclear.

**Methods:**

We retrospectively reviewed 238 patients who underwent anterolateral decompression and instrumentation for single-level thoracolumbar burst fractures from January 2010 and March 2012. The influences of several potential risk factors that might affect supplementary posterior instrumentation were assessed using univariate and multivariate analyses.

**Results:**

Twenty seven patients who developed worsening back pain without neurological deterioration after the anterolateral approach treatment need further posterior instrumentation fixation. The univariate analysis showed that age, disruption of the posterior longitudinal ligament complex (PLC), and fracture level were the risk factors for supplementary posterior instrumentation. However, age and integrity of the PLC were the independent risk factors for supplementary posterior instrumentation by multivariate analyses.

**Conclusions:**

Supplemental posterior instrumentation was necessary in 11.3% of cases following anterolateral decompression and instrumentation in the present study. Older age and disruption of the PLC were the independent risk factors in prediction of supplementary posterior instrumentation in treating thoracolumbar burst fractures.

## Introduction

About 20% of thoracic and lumbar fractures belong to thoracolumbar burst fractures [1,2]. This kind of fracture is frequently associated with neurologic deficits because of encroachment on the neural elements and at times owing to the dynamic nature of the injury.

To some extent, management of thoracolumbar burst fractures is according to clinical and radiographic criteria [3-17]. The purpose of orthopedic surgery includes decompression of the neural elements, restoration of vertebral body height, correction of spinal deformity, and stabilization. Furthermore, surgery can be performed through a posterior approach [18-21] or through an anterolateral retroperitoneal flank approach [22-27], based on the necessity and extent of decompression.

The anterolateral retroperitoneal flank approach allows the surgeon to conduct corpectomy and decompression of the canal. Bone fragments can be withdrawed from the canal under direct vision. After corpectomy, the vertebral column is reconstructed by inserting a prosthesis or graft, restoring height and correcting spinal angulation. When placing anterior instrumentation, the hardware generally incorporates one level above and one level below the fracture.

However, there are about 10% patients who meet failure after anterolateral decompression and stabilization. They need further posterior instrumentation [24,25,27]. Currently, supplementary posterior instrumentation was performed in cases of symptomatic settling and angulation of the spine or instability in spite of anterior instrumentation.

At present, the risk factors for predicting supplementary posterior instrumentation after anterolateral decompression and instrumentation in treating thoracolumbar burst fractures are still unclear. Thus, the purpose of the present study is to identify risk factors that contribute to the need for posterior instrumentation after anterolateral decompression and stabilization for single-level thoracolumbar burst fractures using a multivariate statistical model.

## Materials and methods

### Patients

**Figure 1:**
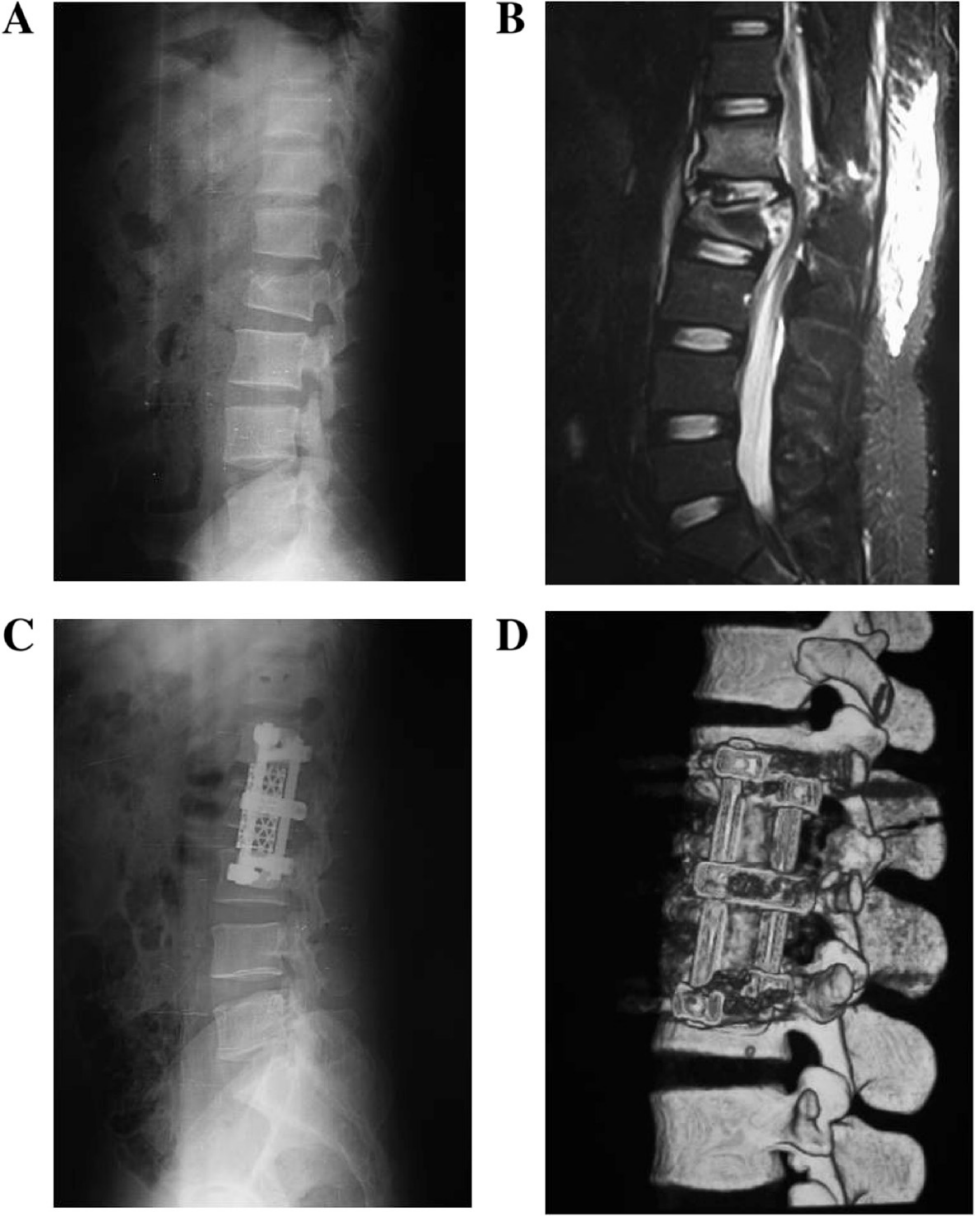
**A 27-year-old man sustained a fall from ground level. (A)** lateral x-ray shows burst fracture of L2. **(B)** MRI shows the burst fracture and canal compromise. **(C)** lateral x-ray and 3D-CT **(D)** show excellent alignment with the rods and screws in place.

**Figure 2:**
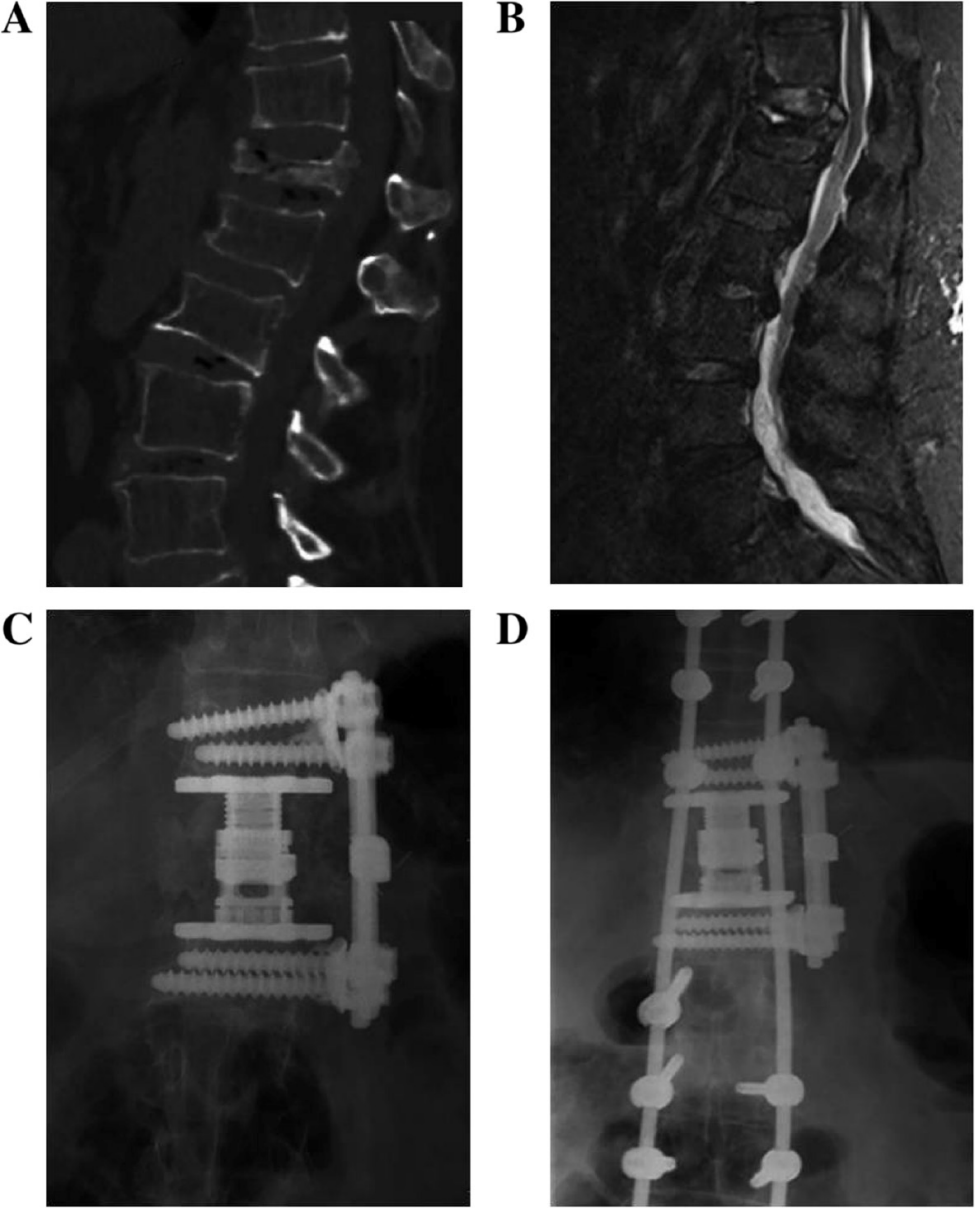
**A 58-year-old woman sustained a fall from ground level. (A)** lateral x-ray shows burst fracture of T12. **(B)** MRI shows the burst fracture and canal compromise. **(C)** AP x-ray show good apposition of the rectangular footplates and the adjacent endplates. One week later the patient was experiencing disabling back pain upon mobilization in thoracolumbar orthosis. Posterior minimally invasive pedicle screws were placed. **(D)** AP x-ray shows stable and satisfactory spinal alignment.

Between January 2010 and March 2012, 238 patients (178 females, 60 males; mean age, 63.2 years; range, 42–87 years) who underwent anterolateral approach and/or posterior approach for single-level thoracolumbar burst fractures at our institution and were followed up for at least 1 year after the procedure were retrospectively enrolled in this study (Figures 1 and 2). The inclusion criterion was that the fractures existed in the anterior and middle columns as described by Denis [2] and fell into groups A3.1–A3.3 of Magerl et al. [28]. The Thoracolumbar Injury Classification and Severity Score (TLICS) [29,30] utilizes the presence or absence of neurological deficit, the integrity of the posterior longitudinal ligament complex (PLC), and the morphology of the fracture (compression, burst, or dislocation). The patients who underwent surgery had a TLICS score of 4 or greater. Exclusion criteria were patients with other types of thoracolumbar fractures, or vertebral fractures above T10, or those patients without follow-up at least 1 year. The study protocol was approved by the local institutional review board and ethics committee. All patients provided written informed consent.

### Technical note

All the anterolateral approaches were performed by the primary spinal surgeon through the left flank according to standard practice [23-26]. After corpectomy, decompression of the thecal sac was ensured from pedicle to pedicle in the axial plane and from the rostral to the caudal intact endplates of the adjacent vertebrae. The anterior column was reconstructed, using iliac autografts or allografts. The carbon fiber-reinforced polymer cages or expandable titanium cages were used. The graft or cage with the largest footplate was consistently selected to reduce subsidence, or telescoping, of the graft or cage into the adjacent vertebral bodies. Cages were packed with artificial graft or autograft harvested from the patient during decompression, supplemented with corticocancellous allograft if necessary. To facilitate graft or cage insertion, distraction was applied on the rostral and caudal bodies through the bicortical screws. Screw length was calculated from axial CT scans before surgery. Overzealous distraction was avoided to prevent screw loosening or pullout. Gentle pressure on the gibbous in a ventral direction was also helpful. The position of the graft/cage was confirmed using both anteroposterior and lateral fluoroscopy. Lateral instrumentation with bicortical screws and dual rods was used in some cases. All patients wore a thoracolumbar clamshell orthosis postoperatively for 3 months.

The integrity of the PLC was evaluated by one of our authors who was blinded to the management or outcomes of the patients. The T1- and T2-weighted images and the short tau inversion recovery (STIR) sequence were used to assess the integrity of PLC consisting of the supra- and infraspinous ligaments, the ligamentum flavum, and the facet capsules [31-36]. Disruption was diagnosed when the black stripe representing the supraspinous ligament was discontinuous. Injury to the infraspinous ligaments was diagnosed with high signal intensity in the interspinous space produced by hemorrhage.

### Potential risk factors

Data regarding age, gender, body mass index (BMI), America Spinal Injury Association (ASIA) impairment scale [37], segmental kyphosis as assessed on preoperative radiography, residual anteroposterior canal diameter as assessed on CT scans, fracture level, fracture age, and surgical approach were collected. ASIA impairment scale was used to assess the neurological deficit on initial examination and at follow-up. Segmental kyphosis was measured on lateral plane radiographs using the angle subtended between the adjacent intact endplates. Residual anteroposterior canal diameter at the injury site was measured from preoperative CT scans and expressed as a percentage of the intact diameter averaged between the rostral and caudal intact canal.

### Statistical analysis

Factors associated with supplementary posterior instrumentation after anterolateral decompression and instrumentation were identified using univariate analysis. The data analysis was performed using SPSS version 19.0 (Chicago, IL, USA).Continuous data were compared between the two groups using the student *t* test, whereas discontinuous data were analyzed using the chi-squared test. All significance tests were two-tailed, with *p* < 0.05 representing statistical significance. In addition, a multivariate logistic regression analysis was performed to identify which independent factors helped predict the supplementary posterior instrumentation after anterolateral decompression and instrumentation in treating thoracolumbar burst fractures.

## Results

### Characteristics of patients

A total of 238 patients with single-level vertebrae cases were finally included. The follow-up is 20.2 ± 8.1 months. Causes of injury were falls in 87, car accidents in 91, vehicle/motorcycle in 19, equestrian in 2, sports in 27, and other causes in 12. L1 was the affected level in 78, followed by T12 in 56, T11 in 51, L2 in 38, and T10 in 15. Surgery was undertaken 10.3 ± 4.4 days following injury, with a length of hospitalization of 17.5 ± 7.7 days. The residual canal on admission measured 45.7% ± 13.9%. Kyphotic angulations on admission, discharge, and last follow-up were 7.1 ± 9.1, 0.4 ± 6.5 (*p* < 0.05), and 1.2 ± 6.6 (*p* < 0.05), respectively (Table 1).

**Table 1:** **Characteristics of patients**

	**Value**
Number of patients	238
Causes of injury	
Fall	87
Car accident	91
Vehicle/motorcycle	19
Equestrian	2
Sports	27
Other causes	12
Level of vertebrae	
T10	15
T11	51
T12	56
L1	78
L2	38
Follow-up	20.2 ± 8.1 mo
Length of hospitalization	17.5 ± 7.7 d
Injury duration before surgery	10.3 ± 4.4 d

*T* thoracic, *L* lumbar, *mo* months, *d* days.

### Characteristics of patients with supplementary posterior fixation

Twenty-seven patients developed worsening back pain without neurological deterioration after the anterolateral approach treatment. Nine patients had neurological deficit on admission, while others were intact. This clinical deterioration was caused by spinal settling and graft migration predominantly into the caudal endplate. Therefore, all of the 27 patients required supplementary posterior fixation, which was undertaken within 13.2 ± 4.5 days after the previous operation. The characteristics of patients with supplementary posterior fixation are presented in Table 2.

**Table 2:** **Results of univariate analysis for supplementary posterior instrumentation in treating thoracolumbar burst fractures**

**Risk factors**	**Number of SPI (** ***n*** **= 27)**	**Number of no SPI (** ***n*** **= 211)**	***p*** **value**
Gender			
Male	7	53	
Female	20	158	0.564
Age	67.1 ± 11.5	57.3 ± 14.5	*0.021*
BMI	29.4 ± 15.5	26.4 ± 8.5	0.712
ASIA in admission	4.1 ± 1.0	4.0 ± 1.0	0.340
ASIA in follow-up	4.5 ± 0.7	4.4 ± 0.7	0.181
Residual canal (%)	43.1 ± 13.4	45.6 ± 14.5	0.091
Angulation in admission	5.6 ± 12.5	7.4 ± 12.1	0.440
Angulation in follow-up	1.5 ± 6.1	1.4 ± 4.2	0.500
Disruption of PLC	19	42	*0.000*
Fracture level			
T10	1	14	
T11	5	46	0.109
T12	5	51	0.337
L1	14	64	*0.015*
L2	2	36	0.260
Kinds of graft			
Autograft	17	145	
Artificial graft	10	66	0.221
Fracture age (days)	12.4 ± 8.1	9.5 ± 4.1	0.081

*SPI* supplementary posterior instrumentation, *PLC* posterior longitudinal ligament complex, *BMI* body mass index, *ASIA* America Spinal Injury Association, *PLC* posterior longitudinal ligament complex, *T* thoracic, *L* lumbar.

### Risk factors by univariate analysis

Univariate analysis was performed to assess risk factors for supplementary posterior instrumentation after anterolateral decompression compared with other patients who were treated with anterolateral instrumentation alone. The results of univariate analysis showed that age, disruption of the PLC, and fracture level were the risk factors for supplementary posterior instrumentation (Table 2).

### Risk factors by multivariate analysis

The associations observed after univariate analysis regarding the potential risk factors enabled the construction of a multivariate logistic regression model for a conjoint analysis to determine which characteristics are independently associated with the supplementary posterior instrumentation. The results of multiple logistic regression analysis are shown in Table 3. Age and integrity of the PLC were the independent risk factors for supplementary posterior instrumentation.

**Table 3:** **Results of multivariate analysis for supplementary posterior instrumentation in treating thoracolumbar burst fractures**

**Risk factors**	**RR**	***p*** **value**
Age	3.44 (1.01–7.77)	0.045
Disruption of PLC	6.44 (1.30–11.76)	0.020
Fracture level	1.24 (0.65–3.57)	0.310

*PLC* posterior longitudinal ligament complex, *RR* risk ratio.

## Discussion

About half of the thoracic and lumbar fractures occur at the thoracolumbar junction (T10–L2), and the majority of these fractures are burst in type involving the anterior and middle columns [3,6,22,25]. The therapeutic options include conservative treatment and surgery. For patients with burst fractures but neurologically intact, conservative treatment may be optimal [15,16]. Surgery is suitable for patients with neurological deficits or persistent pain and for patient whose fractures are deemed unstable with disruption of the posterior ligaments [35,36]. When anterior decompression is deemed unnecessary, posterior instrumentation may be sufficient [18,21]. However, when significant fragmentation of the vertebral body exists and there is poor apposition of the fragments and deformity, anterior grafts and instrumentation are advised [22,26]. Direct access for canal decompression, reconstruction of anterior column, and correction of kyphosis and instrumented fusion with single approach can be achieved by the anterior approach. Moreover, improvement in neurological function has been consistently demonstrated with relatively minimal complications [7,19,26].

In the present study, rods and bicortical screws were used to supple the strut graft and because of the limitations of plates in rigid compared with rods and bicortical screws. Other studies demonstrated the importance of the anterior strut graft by conducting a test that compares three anterior plates and three anterior rods and screws [38-40]. Although the strut graft was performed, settling continues to occur. In the present study, angulation was corrected significantly, from 7.1 ± 9.1 preoperatively to 0.4 ± 6.5 postoperatively, with a slight increase at follow-up to 1.2 ± 6.6 compared to preoperative. The above results are consistent to those of previous studies [22,26,27]. The loss of correction with the passage of time is commonly encountered, well tolerated, and attributed to settling. The more significant increase in angulation with time has been demonstrated in burst fractures when treated nonoperatively [12,13,15].

In the present study, 27 patients underwent supplemental posterior fixation for symptomatic settling of the cage into the superior endplate of the caudal vertebra. These patients did not experience an increase in deficit. Posterior instrumentation fixation was performed within the next few days of the index operation. Of the 27 patients requiring supplemental posterior instrumentation, 19 had PLC disruption compared with other patients who had successful anterior approach fusion. The multivariate analysis demonstrated that the PLC disruption correlated with the need for posterior fixation. The result was consistent with the previous studies that the PLC was of significance in spinal stability. Therefore, rigid posterior fixation is needed in treating thoracolumbar burst fractures.

Another independent risk factor for the need of supplemental posterior instrumentation was age. The mean age of patients needing further posterior fixation was higher than that of the anterolateral group. To some extent, the association of age with the need of supplementary posterior fixation might be attributed to the age-related decrease in bone mineral density [41,42]. However, the measurement of bone mineral density was not conducted in the present study as a routine.

The reoperation rate (11.3%) in the present study was comparable to that of other studies. McAfee et al. [25] conducted Kaneda instrumentation in treating thoracolumbar pathology. Two of 35 cases experienced failure who did need further posterior instrumentation. Kaneda et al. [24] conducted a study about treating thoracolumbar burst fractures by anterolateral Kaneda device. However, pseudoarthrosis was encountered in ten of 150 cases, and further posterior fixation was conducted in ten cases. Sasso et al. [27] performed anterior fusion in treating thoracolumbar burst fractures in 40 patients. Only three patients required additional posterior instrumentation because of disruption of PLC. In summary, different reoperation rates of the need for further posterior instrumentation may be affected by several factors, such as the recruited patients with differences in severity of injury and bone quality.

The limitations of the present study mainly include the following items: (1) Operator expertise and learning curve may be subjective risk factors. We are unable to cancel out the above effects. (2) We did not objectively measure the bone mineral density, which may place an influence on the reoperation rates. Moreover, patient’s comobilities, smoking status, and living condition which have not been evaluated may be all factors that may influence the risk of supplementary posterior instrumentation. (3) The study design, retrospective study, may place bias on the stability of the results.

## Conclusion

In the present study, 27 of 238 patients in which the anterolateral decompression and instrumentation was undertaken in treating single-level thoracolumbar burst fractures did need additional posterior fixation. In univariate analysis, age, disruption of the PLC, and fracture level were the risk factors for further posterior fixation. However, older age and disruption of the PLC were the independent risk factors predicting supplementary posterior instrumentation in treating thoracolumbar burst fractures by multivariate analysis. Further prospective studies are still required to evaluate other potential factors about supplementary posterior instrumentation in treating thoracolumbar burst fractures.
